# Nature and Causes of Diphtheria

**Published:** 1863-05

**Authors:** E. N. Chapman


### NATURE AND CAUSES OF DIPHTHERIA.

The pathology of Diphtheria is not elucidated by autopsies, or by chemical or microscopical examinations. No special structures are invaded, or characteristic lesions discovered; only everywhere is found a dark, grumous blood, filling equally the veins and arteries, and stagnated in various organs. MM. Millard and Peter first pointed out that the blood was of a dirty brown color, resembling liquorice juice or water containing a mixture of soot.

During life there are conclusive proofs, in the malignant cases, of a poisoned, disorganized condition of the circulating fluid—the dark, grumous blood issuing from the tonsils when roughly touched, the spontaneous hæmorrhages, the muscular weakness, the prostrated nerve-power, the clammy sweats, the rapid, soft and shaky pulse, the sphacelation in the fauces, the sequelæ—anæmia, paralysis, etc., the gradual sinking of the patient, and the extinction of life without effort at reaction, or the slightest tokens of constitutional resistance. The evidences of a blood-contamination, equal to those seen in typhus, are infinitely greater than those presented in other diseases now universally conceded to arise from this cause. Chemistry detects no material poison in the air during a diphtheritic epidemic, nor any foreign element in the blood, or any change in its constituents, where patients have died of this disease. The changes, and the agencies producing them, whatever they may be, are inappreciable by this means of investigation.

The enlarged vision afforded by the microscope likewise reveals no sensible alteration in the blood; and the naked eye, which recognizes the fact of the transudation of the liquor sanguinis, and its concretion into a pseudo membrane, gives us equal information with the most powerful glasses. The fibrillæ, granules, pus-cells, etc., that are found, are not distinctive; and the much talk-of algæ are frequently seen on mucous surfaces when covered by morbid secretions. Their ova exist in the atmosphere at all times, but are not developed unless a favorable *nidus* is presented.

From these purely scientific investigations, so rude in comparison with the finer operations taking place in living organisms, we, in this instance, can gain no light, and are obliged, perforce, to learn the true nature of Diphtheria by actual observation in the trial of various modes of treatment. If the free use of stimulants, in the beginning and height of the disorder, subdues the fever, removes the inflammation, causes the membrane to fall, prevents relapses, and, in a word, accomplishes a cure at once rapid and permanent in almost every case where the treatment is commenced early, we must be forced to the conclusion that Diphtheria is a disease of low action—ataxic—and that the inflammation attending it is certainly not idiopathic and active. This is further shown by the greater success following this mode of treatment than any other; and by the significant fact that malignant cases usually escape the *dissolved* state of the blood, and paralytic accidents are, by the same medication, easily remediable.

The cause of Diphtheria is an interesting theme for speculation. There need not be a material agency—a septic poison in the atmosphere—as is the general opinion; which, received into the blood, multiplies itself, like a ferment, and thus contaminates the entire circulating mass; since a change in the normal constituents of the air, or a variation in its electric condition, might render it less adapted to fulfil its part in the transformations constantly going on in the lungs, whence would arise a defect in the vital elaborations of the blood. A faulty state of the atmosphere—one that imperfectly supplied the blood with the influence necessary to its constant renewal —would be scarcely felt by the strong and robust, but would tell with the most effect on the debilitated or those of little vital power. In our experience, the subjects of Diphtheria are, almost universally, children; and when it attacks adults, those of little stamina are singled out, who, at the time, are suffering from unusual exhaustion. Of the former, those inheriting a scrofulous constitution, or any other vicious state of the system, are the ones, as a rule, that are seized. The child has not only to maintain the body *in statu quo*, like the adult, by the constant renewal of the worn-out material, but also to provide for growth and increase. Hence, children inheriting any depravity of the constitution are always pale, debilitated and sickly, and prone to disease; though, should they survive to mature years, they frequently become strong and robust.

That an inappreciable relationship of the atmosphere may render it less fitted to effect the constant changes and renewals of the blood that take place in the air vesicles, and less adapted to elaborate and vitalize it, particularly where there is a defect in the powers of the individual, is more apparent when we recollect that the circulation is the medium for the ingress and egress of all the new and effete materials of nutrition; that constant changes are here going on, affected by living structures; that the blood circulating in its vessels is organized equally with the solids; that in both solids and fluids, by the agency of absorption, assimilation and nutrition are effected. A dtssimilarity between the solids and fluids is apparent, not real. The blood globules or cells are not fixed in one locality, like the cells in the tissues, but are designed, from their office, to float in their nutritive plasma. Hence any noxious material in the blood, or any impediment to its vital transformations, will, equally with changes in solid structures, engender disease. That the cause of Diphtheria is not an animal or vegetable poison, but a state of the air that impairs the vital status of the blood, is further shown by the following considerations: Diphtheria prevails in all seasons and climates, equally in primitive or miasmatic regions, equally in well-cleaned streets, among the better classes, or in courts and alleys among the victims of want and vice; indeed, filth, poverty, vegetable and animal effluvia, do not increase its virulence or cause its dissemination. It is not self-limited, has no fixed stages or increment and decline, may recur several times, does not attack indiscriminately, but as a rule, singles out scrofulous children, or at least individuals whose constitutions are reduced and blood impoverished; and more than all, Diphtheria is not inoculable. M. Trousseau introduced the exudation into his arm and tonsils; and M. Peter inserted it into his lips, rubbed it over his fauces and had it coughed into his eye without any bad result. In fine, we have no evidence that Diphtheria is contagious, or in any way passes from one individual to another. My observation teaches me that, though more than one of a family may be attacked simultaneously, or within a few days of each other, the disease is not communicated to visitors, or to other families in the house. It might be expected that, of a number of children of like organization and habits, breathing the same air and eating at the same table—or, in other words, having similar surroundings—more than one would be attacked at or about the same time. Generally, however, cases of Diphtheria occur here and there in distant localities; whereas, had contagion any influence in the matter, the disease would not be thus limited, or its victims far separated. Diphtheria has undoubtedly occurred at all periods, and in all countries of the world; but from the low intensity of the causation, the disease, except in epidemic seasons, has been confined to isolated cases. Aside from croup, that may owe this origin, many cases of scarlet fever have ended fatally on the appearance of the diphtheritic exudation. With these preliminary observations, I will now give the details of the most interesting cases that have fallen under my observation.—*Prof. E. N. Chapman.*

